# *nfxB* as a Novel Target for Analysis of Mutation Spectra in *Pseudomonas aeruginosa*

**DOI:** 10.1371/journal.pone.0066236

**Published:** 2013-06-07

**Authors:** Mariela R. Monti, Natalia R. Morero, Virginia Miguel, Carlos E. Argaraña

**Affiliations:** Centro de Investigaciones en Química Biológica de Córdoba (CIQUIBIC), CONICET, Departamento de Química Biológica, Facultad de Ciencias Químicas, Universidad Nacional de Córdoba, Ciudad Universitaria, Córdoba, Argentina; University of Illinois at Chicago College of Medicine, United States of America

## Abstract

*nfxB* encodes a negative regulator of the *mexCD-oprJ* genes for drug efflux in the opportunistic pathogen *Pseudomonas aeruginosa*. Inactivating mutations in this transcriptional regulator constitute one of the main mechanisms of resistance to ciprofloxacin (Cip^r^). In this work, we evaluated the use of *nfxB*/Cip^r^ as a new test system to study mutation spectra in *P. aeruginosa*. The analysis of 240 mutations in *nfxB* occurring spontaneously in the wild-type and mutator backgrounds or induced by mutagens showed that *nfxB*/Cip^r^ offers several advantages compared with other mutation detection systems. Identification of *nfxB* mutations was easy since the entire open reading frame and its promoter region were sequenced from the chromosome using a single primer. Mutations detected in *nfxB* included all transitions and transversions, 1-bp deletions and insertions, >1-bp deletions and duplications. The broad mutation spectrum observed in *nfxB* relies on the selection of loss-of-function changes, as we confirmed by generating a structural model of the NfxB repressor and evaluating the significance of each detected mutation. The mutation spectra characterized in the *mutS*, *mutT*, *mutY* and *mutM* mutator backgrounds or induced by the mutagenic agents 2-aminopurine, cisplatin and hydrogen peroxide were in agreement with their predicted mutational specificities. Additionally, this system allowed the analysis of sequence context effects since point mutations occurred at 85 different sites distributed over the entire *nfxB*. Significant hotspots and preferred sequence contexts were observed for spontaneous and mutagen-induced mutation spectra. Finally, we demonstrated the utility of a luminescence-based reporter for identification of *nfxB* mutants previous to sequencing analysis. Thus, the *nfxB*/Cip^r^ system in combination with the luminescent reporter may be a valuable tool for studying mutational processes in *Pseudomonas* spp. wherein the genes encoding the NfxB repressor and the associated efflux pump are conserved.

## Introduction

Mutation is an essential cellular process that contributes to the genetic variability and acts as the driving force of evolution. Several processes are involved in mutagenesis in bacteria, such as erroneous replication and DNA damage by endogenous and exogenous agents, which in turn are tolerated and/or repaired using different mechanisms [1]. Much effort has been devoted to understand these complex processes, which have been preferentially studied in *Escherichia coli.* For example, genetic analysis of mutator and antimutator genes has been used to define the molecular mechanisms of spontaneous mutagenesis in *E. coli* [2]. However, although these processes are evolutionarily well conserved, it has been suggested that there are considerable differences in the mechanisms leading to mutation among bacteria [1].

The genus *Pseudomonas* encompasses one of the most diverse and ecologically significant groups of bacteria [3]. Members of this genus are capable of thriving in highly diverse ecological niches due to their versatile metabolic capacity and broad potential for adaptation to fluctuating environmental conditions. This unique feature of *Pseudomonas* spp. implies a remarkable degree of genomic diversity and genetic adaptability to customize its genome to fit the requirements for survival in diverse niches [4]. In this regard, chronic airway infection by *P. aeruginosa* is the best studied adaptation to a natural environment. This microorganism causes severe acute nosocomial-acquired infections in immunocompromised patients, and it significantly contributes to the development and progression of the chronic pulmonary disease in cystic fibrosis (CF) patients [5]. Long-term persistence in the lung environment involves physiological changes [6] and an intense genetic adaptation frequently achieved by loss-of-function mutations in global regulatory genes that leads to an enhanced antimicrobial resistance, altered virulence and specific metabolic adaptation [7]. It has been proposed that these genetic changes are caused by exposure to the oxidative stress prevailing in the CF lung [8]. In addition, a relevant role of mutator strains in the genetic adaptation of *P. aeruginosa* to the airways has been suggested in several studies [9]. These mutator strains show defects in the DNA mismatch repair system (MMR-; *mutS*, *mutL* or *uvrD* genes) [10] or the DNA oxidative lesions repair system (GO-; *mutT*, *mutY* or *mutM* genes) [11]. Nevertheless, the molecular mechanisms contributing to mutagenesis and adaptation of *P. aeruginosa* are poorly understood to date.

Mutation detection systems are a very important tool to elucidate the underlying molecular events leading to mutagenesis in bacteria. At present, a limited number of test systems are available to analyze mutagenic processes in *Pseudomonas* spp. For example, a plasmid-based system to study mutations in *P. putida* relies on the transcriptional activation of the promoterless phenol degradation operon *pheBA* [12]. The phenol-utilizing mutants mainly accumulate the GC>TA transvertion, although deletions from 2-bp to 23-bp, and transposition of the mobile DNA elements Tn*4652* and IS*1411* are also detected, which result in the creation of a sequence similar to the σ^70^-specific promoter consensus for transcription of the *pheBA* operon. In addition, a set of plasmids containing a silent *pheA* gene was developed for selection of revertant *P. putida* cells able to grow on phenol [13]. Different base substitutions and a 1-bp deletion within *pheA* are detected using these plasmids. A GFP-based mutation detection plasmid tests the reversion of a 1-bp insertion in *P. aeruginosa* [14]. The *rpoB* gene was used to characterize spontaneous mutations in the chromosome of *P. aeruginosa* and *P. putida* [15]. The highly conserved *rpoB* gene, that encodes the β subunit of RNA polymerase, is the target of mutations leading to rifampicin resistance (Rif^r^) in both *Pseudomonas* spp. The *rpoB*/Rif^r^ system senses base substitutions that cause amino acid changes in the central rifampicin binding pocket (cluster I–III) or the N-terminal cluster in RNA polymerase. Finally, a new test system based on the transcriptional control of the phenol degradation genes *pheBA* by the *E. coli* P*_tac_* promoter and the LacI repressor was employed in *P. putida* [16]. The phe-lacI system is randomly inserted into the chromosome allowing detection of different mutations that inactivate *lacI* or alter the *lac* operator sequence.

In the present work, we evaluated the use of the chromosomal *nfxB* gene as a new target for analyzing mutation spectra in *P. aeruginosa*. *nfxB*, which is located upstream of *mexCD*-*oprJ* but divergently transcribed from this operon, encodes a transcriptional repressor that regulates MexCD-OprJ and its own expression by binding to the *nfxB*-*mexC* intergenic region [17], [18]. Previous studies suggested that mutations within *nfxB* impair or abolish the NfxB repressor activity leading to overexpression of the MexCD-OprJ efflux pump and resistance to fluoroquinolone antibiotics such as ciprofloxacin (Cip^r^) [17]–[19]. To validate the *nfxB*/Cip^r^ system, we sequenced *nfxB* in 240 Cip^r^ mutants derived from the wild-type and mutator strains or treatment with mutagenic agents. A broad range of mutations was observed in *nfxB* including all base substitutions, 1-bp deletions and insertions, >1-pb deletions and duplications. Localization of mutated residues on a tridimensional structural model of NfxB generated *ad-hoc* and analysis of the plausible consequences on protein structure/function supported a loss-of-function effect for these mutations. In addition, the *nfxB* mutation spectra observed in different mutator backgrounds or induced by mutagenic agents were consistent with the previously characterized mutational specificities. Finally, we demonstrated the efficacy of a luminescence-based reporter to detect *nfxB* mutants previous to the sequencing analysis.

## Results

### Sequence analysis of *nfxB* in ciprofloxacin resistant clones

**Figure 1:**
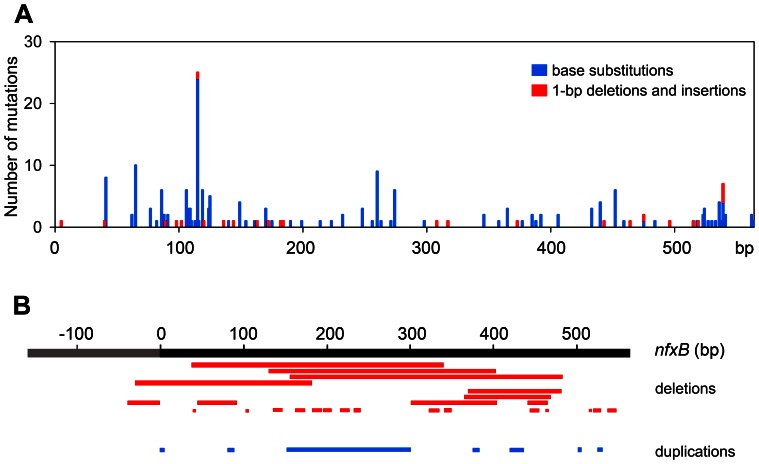
Distribution of mutations in *nfxB*. The position of all mutations detected in *nfxB* among 240 Cip^r^ clones derived independently from different genetic backgrounds or treatment with mutagens is plotted. (A) The number of base substitutions (blue bars) and 1-bp deletions and insertions (red bars) detected at each nucleotide position of the open reading frame is shown. (B) >1-bp deletions (red bars) and duplications (blue bars) are displayed over the open reading frame (black bar) and promoter region (grey bar) of *nfxB*.

In order to evaluate the use of *nfxB* as a new target to analyze mutation spectra, we screened a large collection (240) of ciprofloxacin resistant (Cip^r^) clones derived independently from different genetic backgrounds (wild-type, *mutS*, *mutT*, *mutY* and *mutM*) or treatment with mutagens (2-aminopurine, cisplatin or hydrogen peroxide). PCR amplification and further sequencing of the entire open reading frame (564-bp) and promoter region (160-pb) of *nfxB* from these Cip^r^ clones revealed that different mutations occurred within this gene including each of the six base substitutions, 1-bp deletions and insertions, >1-pb deletions and duplications (Table S1). We detected 71 base substitutions in 62 different sites (Table S1), which were distributed over the entire gene with preference at regions spanning from 100-bp to 125-bp and 525-bp to 550-bp (Figure 1A). Transitions and transversions occurred at 37 and 32 different sites, respectively (Table S1). Deletions and insertions of 1-bp were observed in 23 distinct positions that were distributed across *nfxB* more homogenously than base substitutions (Figure 1A). In addition, we detected deletions and duplications ranging from 2-bp to 302-pb (Table S1). Both mutation types involved nucleotides spanning the entire gene without any preference for a region (Figure 1B). These results demonstrate that the *nfxB*/Cip^r^ system senses a broad range of mutations.

### Mapping of changes on the predicted NfxB structure

Among the *nfxB* mutations reported in this work (Table S1), base substitutions generated missense mutations (70%) and nonsense mutations (27%) that changed 19 different amino acid-coding codons to premature stop codons. Additionally, we observed two base substitutions that changed the stop codon to an amino-acid-coding codon, which may add 67 amino acids to the C-terminal of NfxB. 1-bp deletions and insertions produced frameshifts that generated premature stop codons in some cases. Deletions and duplications of >1-bp removed or added amino acids to NfxB and, eventually, produced frameshifts.

**Figure 2:**
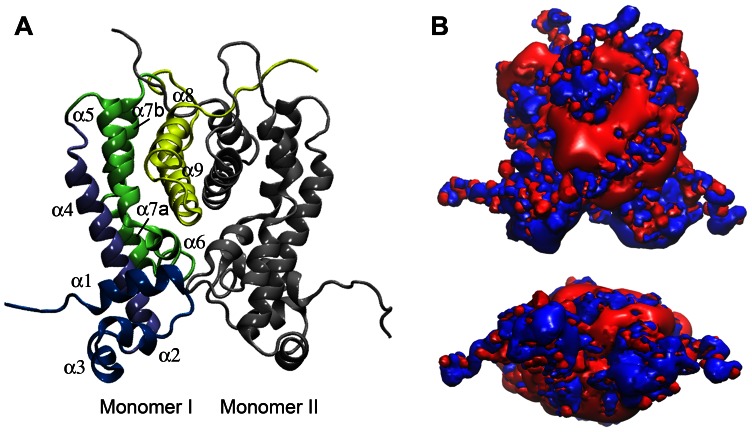
3D-structural model of NfxB. (A) Ribbon representation of the homology-modeled NfxB dimer based on the TetR-like transcriptional repressor LfrR. Helices α1–α3 are shown in blue, α4 in light blue, α5–α7b in green, and α8 and α9 are in yellow. N-terminal helices α1 to α3 and C-terminal helices α8 and α9 corresponded to LfrR helices involved in DNA-binding and dimerization, respectively. (B) Front (above) and top-down (below) visualization of electrostatic potential distribution in the NfxB dimer as determined using APBS. Positive and negative potentials are indicated in blue and red, respectively. Note that a large positive charged surface is located on the N-terminal loop between helices α1 and α2 and helices α2 and α3, which could form the putative DNA-binding channel in the NfxB dimer.

**Figure 3:**
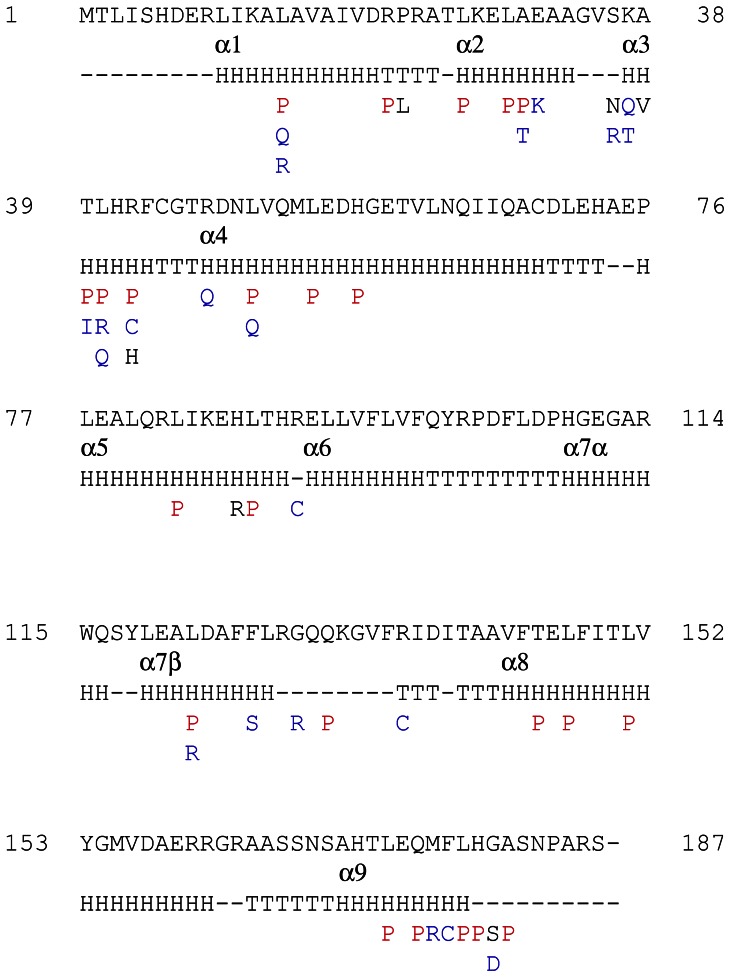
Amino acid substitutions in NfxB. Amino acid changes in NfxB caused by missense mutations are indicated with the one letter nomenclature. Amino acid substitutions for proline are shown in red, and changes to residues that differ in polarity or charge from the original one are depicted in blue. The secondary structure obtained from the NfxB homology model is shown (Nomenclature H: Helix; T: Turn; -: Coil). The nine α-helices are numbered from α1 to α9.

To assess the localization and possible functional consequences of missense mutations, and since there is not structural data for NfxB to date, we generated a 3D-structural model of this repressor (Figure 2A). The NfxB model was obtained by homology modeling with MODELLER [20] using the tetrameric crystallographic structure of the TetR-like transcriptional repressor LfrR (PDB code: 2V57) [21]. Although there are no evidences of the NfxB oligomeric state at present, we show the dimeric model since it is expected that the dimer is the minimal unit able to form the DNA-binding channel (Figure 2). The predicted structure of the NfxB monomer displayed an almost entirely α-helical architecture with 63% of residues located in α-helices (Figure 2A and 3). The secondary structure of NfxB predicted using JPred [22] also showed a high proportion (70%) of residues in α-helices whose positions were comparable to that showed in the model (Figure S1). Nine α-helices were identified in the NfxB model (Figure 2A). The C-terminal helices α4 to α8 were arranged in three layers, wherein helices α8 and α9 (residues V143 to L178) corresponded to the LfrR C-terminal helices involved in protein dimerization [21]. In addition, a large positive charged surface was visualized on the N-terminal loop between helices α1 and α2 and the helices α2 and (3 (residues R21 to F43) when the electrostatic properties of NfxB were determined using APBS [23] (Figure 2B). This correlated with the localization of the DNA-binding domain in the N-terminal helices of LfrR [21]. Thus, the N-terminal region spanning residues R21 to F43 could form the putative DNA-binding channel in the NfxB dimer.

Based on the NfxB model, we determined that 40% and 24% of missense mutations affected residues located at the putative DNA-binding channel and dimerization surface, respectively (Figure 3). Furthermore, it was possible to distinguish groups of variants on the basis of their effect on structure or function. The first one, which accounts for 46% of missense mutations, corresponded to substitutions of different amino acids for proline (Table S1). A high proportion (83%) of these mutations fell into predicted α-helices (Figure 3). Since proline acts as a structural disruptor of regular secondary structure elements such as α-helices [24], these changes could be altering protein folding or structure. The other group (42% of missense mutations) involved changes to residues that differ in polarity or charge from the original one (Figure 3 and Table S1). These mutations mainly affected residues forming the probable DNA-binding channel, and thus, they could alter NfxB electrostatic properties and DNA binding. All together, these data support that the detected mutations in *nfxB* may produce loss-of-function changes in the NfxB repressor.

### Characterization of spontaneous and mutagen-induced mutation spectra in different genetic backgrounds using the *nfxB*/Cip^r^ system

#### I- Wild type strain

**Figure 4:**
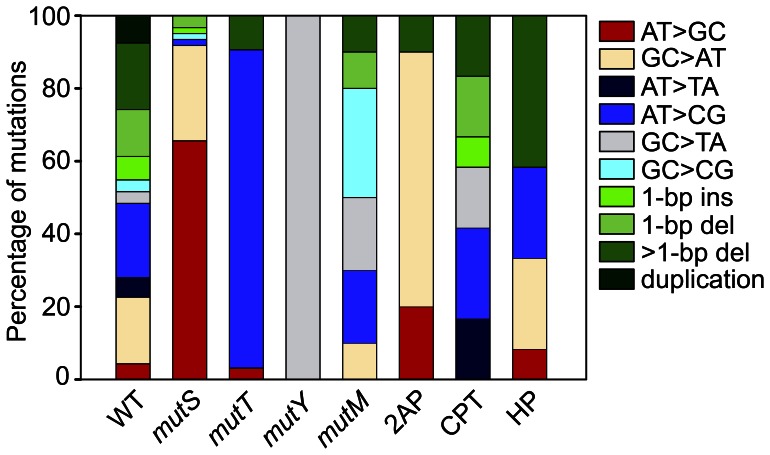
Spontaneous and mutagen-induced mutations in *nfxB*. The *nfxB* mutations detected in the wild-type (WT) and mutator strains (*mutS*, *mutT*, *mutY* and *mutM*), and the WT strain after exposure to the mutagens 2-aminopurine (2AP), cisplatin (CPT) and hydrogen peroxide (HP) are summarized. The percentage of each mutation type relative to the total number of mutations detected for each strain or treatment is plotted using a color code.

We analyzed 93 spontaneous mutations isolated from the wild-type (WT) strain PAO1 (Table S1). The mutation spectrum was composed of all base substitutions (53%), 1-bp insertions and deletions (19%), >1-bp deletions (18%) and duplications (8%) (Figure 4 and Table S2). Among base substitutions, the GC>AT transition and the AT>CG transversion were mainly detected. Base substitutions occurred in 21 different sites, wherein nucleotide 115 represented a significant hotspot (*p*-value<0.05) comprising 35% of all base substitutions (Table S1). This mutation hotspot showed exclusively the AT>CG transition. The most mutable dinucleotides were CA and AC, which was due to the presence of the hotspot at nucleotide 115 within the C**A**C sequence (the mutated nucleotide is indicated in bold, Tables S1 and S3). Insertions and deletions of 1-bp were detected in 16 different positions located within mononucleotide runs, as it has frequently been reported in previous works, and outside of runs (Table S1). Deletions and duplications of different sizes (2-bp to 302-bp) were observed, however small ones (<15-bp) were most frequently detected (Table S1).

#### II- Mutator strains

To test the accuracy of the *nfxB*/Cip^r^ system for detecting the mutational specificities displayed by different mutator strains, we analyzed the *nfxB* mutation spectrum in MMR- and GO-deficient strains. First, mutation rates per replication to Cip resistance were calculated for each mutator strain (Table 1). When compared to the WT strain, mutation rates of the *mutS* and *mutT* strains were greatly increased whereas the *mutY* and *mutM* strains showed a mild mutator phenotype. Then, *nfxB* was sequenced from Cip^r^ colonies derived from each mutator strain (Table S1). For the *mutS* and *mutT* strains, a luminescence-based reporter was employed to identify *nfxB* mutants since a proportion of the Cip^r^ colonies were mutated in *nfxB* (see below).

**Table 1:** Mutation rates to ciprofloxacin resistance^a^.

Strain	Mutagen	*µ* (CL)	Normalized rate^b^
WT^b^	–	4.3 (2.3–7.2) ×10^−10^	1
*mut*S	–	3.4 (2.5–4.6) ×10^−6^	7907
*mut*T	–	4.6 (4.0–5.0) ×10^−6^	10698
*mut*Y	–	2.0 (1.0–3.2) ×10^−8^	47
*mut*M	–	9.3 (3.6–16.4) ×10^−9^	22
WT^b^	–	1.8 (1.1–2.7) ×10^−9^	1
WT	2AP	1.9 (1.4–2.5) ×10^−8^	11
WT	CPT	2.8 (1.7–4.3) ×10^−9^	2
WT	HP	2.0 (1.3–2.8) ×10^−9^	1

a Mutation rates (*µ*) per replication and 95% confidence limits (CL) were calculated as described in Material and Methods. Mutation rates for the WT and mutator (*mutS*, *mutT*, *mutY* and *mutM*) strains were calculated at 1.0 µg/ml Cip. Assays with 2-aminopurine (2AP), cisplatin (CPT) and hydrogen peroxide (HP) were performed at 0.5 µg/ml Cip. ^b^Mutation rates calculated for the WT strain at each set of experiment (mutator strain and mutagen assessment) were used for data normalization.

In the *mutS* background, transitions were dominant accounting for 92% of mutations (Figure 4 and Table S2). The AT>GC transition was preferentially promoted in this genetic background since it was increased ∼17-fold relative to the WT strain. Mutation hotspots were observed at nucleotides 86 and 260 (*p*-value<0.05), which only showed the AT>GC transition. The CT and TG dinucleotides were most frequently mutated, as determined using iMARS [25] (Table S3). Both dinucleotides were almost exclusively present in sites undergoing the AT>GC transition. Visual inspection of sequences surrounding the mutated nucleotide revealed that this mutation occurred in 14 different sites of *nfxB*, where 9 had the C**T**G trinucleotide (which included the CT and TG dinucleotides; the mutated nucleotide is indicated in bold) (Table S1). Conversely, AT>GC transitions were not observed in CTG sequences in the WT strain (Table S1). In the *mutT* strain, the AT>CG transversion was almost exclusively observed comprising 88% of all mutations (Figure 4 and Table S2). The frequency of this transversion was increased ∼4-fold when compared to that observed in the WT strain. The majority (89%) of AT>CG occurred in CA, AG and AA dinucleotides at 10 different sites within *nfxB* (Tables S1 and S3). This transversion was detected in CA dinucleotides in the WT strain, but it almost exclusively occurred at nucleotide 115 (Table S1). The GC>TA transversion was the unique mutation detected in the *mutY* strain, which was increased ∼33-fold in this genetic background relative to the WT strain (Figure 4 and Table S2). 80% of this transversion occurred in the **G**A dinucleotide (the mutated nucleotide is indicated in bold; Table S1). Transversions dominated the mutation spectrum in the *mutM* strain (Figure 4 and Table S2). The AT>CG, GC>TA and GC>CG mutations were detected in 70% of the Cip^r^ clones isolated from this mutator strain. Among these transversions, GC>TA and GC>CG were promoted since they were increased ∼7- and ∼10-fold, respectively, over the WT level.

#### III- Wild type strain exposed to mutagenic agents

*P. aeruginosa* WT cells treated with the base analog 2-aminopurine (2AP) showed a modest increment of mutation (Table 1). Conversely, treatment with cisplatin (CPT) or hydrogen peroxide (HP) had no effect on the mutation rate of the WT strain (Table 1). Cell exposure to 2AP resulted in a shift in the mutation spectrum to transitions (90%) (Figure 4 and Table S2). The AT>GC and GC>AT mutations were increased ∼5- and ∼4-fold upon 2AP treatment. A high proportion (86%) of the GC>AT transitions occurred in the C**C** dinucleotide whereas AT>GC was detected in the C**T**G trinucleotide (the mutated nucleotide is indicated in bold; Table S1). Although CPT and HP did not produce an increase of mutation rates at the tested concentrations, both mutagens induced changes in the nature of mutations. The AT>TA, AT>CG and GC>TA base substitutions, 1-bp insertions and deletions, and >1-bp deletions were detected in CPT exposed cells (Figure 4 and Table S2). When compared with the mutation spectrum observed in the WT strain, AT>TA and GC>TA were increased ∼3- and ∼6-fold, respectively. Base substitutions (86%) were mainly present at sites involving adjacent G and A nucleotides (Table S1). The sequence context preferences observed in the 2AP- and CPT-induced spectra were not detected in the spontaneous spectrum of the WT strain (Table S1). In cells treated with HP, the *nfxB* mutation spectrum mainly included GC>AT and AT>CG, and >1-bp deletions (Figure 4 and Table S2). Deletions were increased from 18% to 42% after exposure to HP.

In conclusion, the mutation spectrum characterized for each mutator background and mutagen treatment was notably different from that observed in the WT strain. In addition, the observed changes were consistent with the mutational specificities previously reported (see Discussion) indicating that the *nfxB*/Cip^r^ system is able to accurately sense modifications of the mutagenic processes occurring in *P. aeruginosa* cells.

### A luminescence-based reporter for detecting *nfxB* mutants

**Figure 5:**
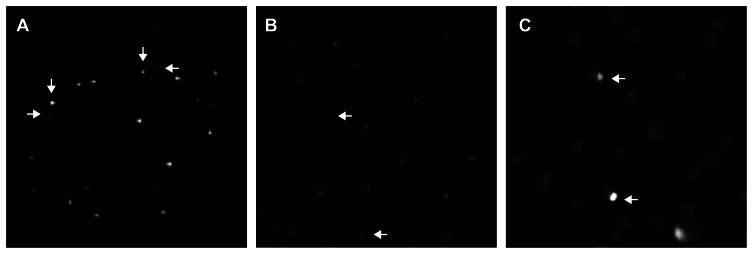
Identification of *nfxB* mutants using a luminescence-based reporter. (A) Colonies of the *mutT* strain selected at 1.0 µg/mL Cip after 48 h of incubation. Examples of colonies with basal (non-mutated) and increased (*nfxB* mutants) luminescence are indicated (horizontal and vertical arrows, respectively). (B–C) Colonies of the *mutT* strain selected at a subinhibitory Cip concentration (0.4 µg/mL) after 24 h (B) and 56 h (C) of incubation. Detection of a subpopulation of cells with increased luminescence arising from colonies with basal luminescence indicates the emergence of *nfxB* mutants (examples are indicated by arrows).

The molecular bases of resistance to Cip in *P. aeruginosa* are mutations in *gyrA an*d *parC*, that encode DNA gyrase and DNA topoisomerase IV, respectively, and mutations in genes encoding the transcriptional regulators of multidrug efflux pumps like *nfxB* [26]. We demonstrated in a previous study that different resistance mutations are observed in Cip^r^ subpopulations of the *mutS* and *mutT* strains by sequencing *gyrA*, *parC* and *nfxB* [27]. A fraction of these Cip^r^ subpopulations corresponds to *nfxB* mutants, which varied with the Cip concentration used for selection. In the present work, we used a chromosomal luminescence-based reporter for identification of *nfxB* mutants prior to sequencing analysis. This reporter, previously validated in *P. aeruginosa*, was achieved by fusing the *mexC* promoter to the *luxCDABE* operon present in the pUC18-mini-Tn7T-Gm-*lux* delivery transposon vector that integrates this fusion at the attTn7 chromosomal site (see Materials and Methods) [27], [28]. Thus, the *luxCDABE* operon is normally repressed at a basal level by endogenous NfxB. Loss-of-function mutations in *nfxB* induce *luxCDABE* expression, which allows detection of *nfxB* mutants by the acquisition of an increased luminescent phenotype [27]. For the *mutT* strain, 43% and 70% of colonies with increased luminescence were observed at 0.5 and 1.0 µg/ml Cip, respectively (Figure 5A). At these Cip concentrations, the percentage of colonies with increased luminescence was 30% and 10% for the *mutS* strain. All selected colonies exhibiting an increased luminescence showed a stable phenotype and were mutated in *nfxB*, as revealed by sequencing analysis (61 and 32 colonies analyzed for *mutS* and *mutT*, respectively) (Table S1). Conversely, *nfxB* was not mutated in colonies showing a background luminescence (10 colonies analyzed for each mutator strain). When we inserted the luminescent reporter into the chromosome of the WT strain (selected at 0.5 and 1.0 µg/ml Cip, and at 0.5 µg/ml Cip after exposure to mutagens) and *mutY* and *mutM* mutators (selected at 1.0 µg/ml Cip), 100% of the Cip^r^ clones exhibited an increased luminescence phenotype (data not shown). This was consistent with the fact that all Cip^r^ colonies selected for sequencing analysis were mutated in *nfxB* (Table S1).

The luminescent reporter was used in our previous study to analyze the emergence of *nfxB* mutants during cell growth in solid media containing sub-inhibitory Cip concentrations (0.06 µg/ml for the WT strain, and 0.4 µg/ml for the *mutS* and *mutT* strains) [27]. ∼0.06% and 0.3% of *nfxB* mutants emerged in WT and mutator populations, respectively [27]. For example, exposure of *mutT* cells to 0.4 µg/ml Cip induced the emergence of colonies with an increased luminescence after 56h of incubation, which were not previously observed at 24h of incubation (Figure 5B and 5C). This increased luminescence was detected in a small external sector of the colonies (see arrows in Figure 5C). When these mixed colonies were further isolated on LB plates, two distinct populations of cells with either low or high luminescence were observed. Sequence analysis indicated that only the most luminescent population was mutated in *nfxB* (data not shown). Colonies with an increased luminescence were not observed in *mutT* cells in the absence of Cip at the tested conditions (data not shown). Stimulation of *nfxB* mutagenesis at sub-inhibitory Cip levels may be produced by a transient increase in the mutation rate induced by this antibiotic [27]. This mutagenic effect has been previously reported for fluoroquinolones and other antibiotics in different bacterial species [29]. Different mechanisms has been proposed for induction of mutagenesis such as DNA oxidation due to an oxidative burst, mutagenic repair of DNA damage by recombination, induction of error-prone polymerases mediated by the SOS response or direct interaction with DNA [29].

We demonstrated that this luminescent reporter may be used to detect subpopulations of *nfxB* mutants, as showed for the *mutS* and *mutT* strains, and to follow the emergence of *nfxB* mutants over time.

## Discussion

Understanding molecular mechanisms of mutational processes requires test systems for monitoring mutation rates and analyzing mutation spectra. Several mutation detection systems have been developed for bacteria, however the majority are well suited for use in *E. coli*. In recent years, studies have been focused on mutational mechanisms in other bacteria such as the diverse and ecologically significant *Pseudomonas* genus [30]. There are a limited number of test systems for analyzing mutations in *Pseudomonas* spp. Moreover, most of these systems are based on the selection of mutants capable of growing in phenol [12], [13], [16], with exception of the *rpoB*/Rif^r^ and *gfp* systems [14], [15], and have been applied to studies of mutational processes in *P. putida*.

In the present report, we evaluated the use of *nfxB* as a new target to analyze mutation spectra in *P. aeruginosa*. Inactivating mutations in this gene constitute one of the main mechanisms leading to ciprofloxacin resistance (Cip^r^) in this opportunistic pathogen [17]–[19]. We demonstrated that the *nfxB*/Cip^r^ system exhibits several advantages compared with the previously characterized systems. A simple and unambiguous assignment of mutations was achieved since the complete open reading frame and the promoter regions of *nfxB* are sequenced using a single primer. Conversely, mutations in *rpoB* map into clusters I-III or the N-terminal cluster, and consequently two sequencing reads are needed to determine mutational sites [15]. In the pheA-lacI system, 5 primers are used to cover the complete *lacI* and *lac* operator sequences wherein mutations are detected [16]. *nfxB* allowed the detection of mutations occurring in the *P. aeruginosa* chromosome. Previous studies in *E. coli* have demonstrated that mutational processes may operate differently in the chromosome than extrachromosomal elements under some conditions [31], [32]. Therefore, even though this has not been studied in *Pseudomonas*, it is important to consider this aspect when a mutational analysis is carried out. The fact that *nfxB* is an endogenous gene simplifies the analysis of mutations. For example, in the pheA-lacI and plasmid-based systems is necessary to introduce these elements before selection of mutants. In addition, it is important to examine if random insertion of the pheA-lacI system into the chromosome does not alter genes involved in mutational processes.

The *nfxB*/Cip^r^ system detected different mutation types that comprised all base substitutions, 1-bp deletions and insertions, and deletions and duplications ranging from 2- to 302-bp. To compare this system with the *rpoB*/Rif^r^ system, we sequenced *rpoB* in 44 rifampicin resistant clones isolated from the wild-type (WT) strain PAO1 used in this work (Table S4). As previously reported [15], the spontaneous *rpoB* mutation spectrum included almost exclusively three of the six base substitutions: AT>GC, GC>AT and AT>TA. Therefore, and differently from *rpoB* that is well suited for analyzing base substitutions, *nfxB* is able to detect a broad range of mutations in the chromosome of *P. aeruginosa*. This results from selection of loss-of-function changes in the NfxB repressor. Based on our 3D-structural model of NfxB, missense mutations mainly affected residues located at the putative DNA-binding channel and dimerization surface. Additionally, we predicted two probable effects of these mutations on NfxB: alteration of its structure by substitutions of different amino acids for the structural disruptor proline into predicted α-helices, and its DNA-binding activity by changing the polarity or charge of residues forming the putative DNA-binding channel. Amino acid substitutions in NfxB of *P. aeruginosa* linked to multidrug resistance phenotypes and eventually to overexpression of MexCD-OprJ have been previously reported: R21H [33], [34]; A30T [35]; R42G [36]; R42H [37]; R42C [33]; D56G [33], [34]; L62V [35]; R82L [38]; H87R [39]; Y109H [34]; K132I [33]; K132R [33]; M155R [34] and N183I [33]. Most of these mutations are positioned in the putative DNA-binding and dimerization regions predicted in our NfxB model. Thus, we described inactivating mutations in 33 additional residues of NfxB.

Different mutagenic processes were detected using the *nfxB*/Cip^r^ system since the mutation spectra characterized for each mutator background and mutagen exposure corresponded to its recognized mutational specificity. In the *mutS*-deficient strain, mutations were almost exclusively transitions and AT>GC was preferentially promoted as reported in previous studies using *rpoB* and *lacI* targets in *E. coli* [40], [41]. Mutations indentified in GO-deficient backgrounds correlated with the lack of 8-oxodeoxyguanine (8-oxoG) repair. The *mutT* strain exhibited an increase of the AT>CG transversion, which is consistent with a deficiency of 8-oxoG hydrolysis in nucleotide pools by MutT [42], [43]. *mutM* and *mutY* mutator strains showed specific GC>TA increases as expected from the role of MutM and MutY in the repair of 8-oxoG present in double-stranded DNA [43], [44]. A prevalence of AT>CG and GC>TA was also detected in the mutation spectra of *rpoB* from *mutT*- and *mutYM*-deficient strains of *E. coli*, respectively [40]. These mutations were not observed in *rpoB* from *mutM* and *mutT* mutator strains of *P. aeruginosa* [45] indicating that the *rpoB*/Rif^r^ system may not be suitable to sense mutations related to the deficiency of *mutM* and *mutT* in *P. aeruginosa.* Additionally, the GC>CG transversion was also promoted in the *mutM* strain. This base substitution was induced by oxidative stress in *rpoB* and *lacI* from *mutM*-deficient strains of *P. aeruginosa* and *E. coli*, respectively [45], [46]. The base analog 2AP causes AT>GC and GC>TA transitions by mispairing with C [47]. As predicted, both mutations were increased in the WT strain after exposure to this mutagen. This was not observed in *rpoB* of *E. coli* where only GC>TA was induced by 2AP [40]. Treatment with cisplatin (CPT) produced an increase of AT>TA and GC>TA transversions in the *nfxB* mutation spectrum from the WT strain. As showed in previous studies, both mutations were also induced by this mutagen in *E. coli* cells [40], [48], [49]. Hydrogen peroxide (HP) increased the frequency of deletions involving a small number of nucleotides (<15-bp) in *P. aeruginosa*. A previous work demonstrated that exposure of *E. coli* cells to HP also promoted the occurrence of deletions ranging from 2- to 10-bp, however, and differently to that observed in *nfxB*, these mutations were detected within dinucleotide repetitive sequences [50].

Most of test systems employed in *Pseudomonas* sense mutations in a limited number of surrounding sequence contexts. Systems based on the reversion of *pheA* or *gfp* only use one site to monitor base substitutions and a 1-bp deletion [13], [14]. Base substitutions occur in two different sites to activate the transcription of the promoterless operon *pheBA* [12] and in 24 sites of *rpoB* [15]. Changes in 40 sites within *lacI* and the *lac* operator are detected using the pheA-lacI system [16]. On the other hand, numerous sequence contexts can be analyzed using *nfxB*. We detected 71 base substitutions in 62 different sites, and deletions and insertions of 1-bp distributed among 23 distinct positions. This allowed us to identify mutation hotspots and preferred sequence contexts. In the spontaneous mutation spectrum from the WT strain, a hotspot was identified at nucleotide 115 that showed exclusively the AT>CG transversion. Two AT>GC hotspots were observed at nucleotides 86 and 260 in the *mutS* background. In addition, we found that AT>GC transitions were predominantly detected in the C**T**G trinucleotide (the mutated nucleotide is indicated in bold). The hotspot at nucleotide 86 showed this sequence context and the hotspot at nucleotide 260 occurred at a C**A**C trinucleotide (or G**T**G in the other strand). Thus, AT>GC mutations were favored at **T**G sequences (where T had a 3′-G). Similarly, although AT>GC changes predominantly occur at G**T** (where T had a 5'-G) sequences, **T**G sequences are also preferred in *rpoB* and *lacI* from *mutS*-deficient strains of *E. coli* [40], [41]. AT>CG was almost exclusively observed in C**A**, **A**G and **AA** dinucleotides in the *mutT* strain, and GC>TA mainly occurred in **G**A sequences in the *mutY* strain. A preference for these sequence contexts are also observed in *rpoB* from *mutT*- and *mutYM*-deficient strains of *E. coli* [40]. For the base analog 2AP, we showed that GC>AT transitions mainly occurred in C**C** dinucleotides. This is not in agreement with previous works showing that a C preceded by a G is the preferred sequence context for GC>AT transitions in *E. coli* cells exposed to 2AP [40], [51]. Base substitutions observed in WT cells treated with CPT were present at sites involving adjacent G and A nucleotides. The mutagenic agent CPT mainly promotes 1,2-intrastrand cross-links at ApG and GpG sites causing different base substitutions at these sites [49], [52]. Accordingly, the primary source of CPT-induced mutations was the ApG adduct in *nfxB*. In addition, our results showing that CPT induced AT>TA at AG sites and GC>TA at GA sites by mutating the 5′-nucleotide of the adduct correlate with previous findings [40], [49], [52].

Along with the advantages demonstrated for the *nfxB*/Cip^r^ system, we also showed the utility of a luminescence-based reporter for identification of *nfxB* mutants. In this work, we used this reporter to detect *nfxB* mutants derived from the *mutS* and *mutT* strains previous to sequencing analysis since a fraction of both Cip^r^ subpopulations was mutated in this gene. Thus, the luminescent reporter should be used in bacterial strains or conditions wherein resistance to Cip is mediated by mutations in different target genes. For these cases, our luminescent reporter may be applied as it is contained in a broad-range mini-Tn7 transposon that has been widely used for chromosomal insertion in *Pseudomonas* and other bacterial species [28]. In addition, the luminescent reporter was used to monitor the emergence of *nfxB* mutants in cells exposed to sub-inhibitory Cip levels over time (results from this work and Morero *et al*. 2011). Similarly, it could be also employed to follow other stress-induced mutagenic processes such as those occurring in stationary phase or upon starvation conditions.

Finally, the use of *nfxB*/Cip^r^ as a test system could be extended in a straightforward manner to other *P. aeruginosa* strains and probably to other *Pseudomonas* spp. We found at least 19 putative orthologous genes to PAO1 *nfxB* among other *P. aeruginosa* strains and species of this genus. Among them, all orthologs found in *P. aeruginosa* strains were associated to *mexCD*-*oprJ* homologue genes in the genome and their function in antibiotic efflux was experimentally demonstrated for some strains (e.g. LESB58 and UCBPP-PA14 strains). Although functional information about *nfxB* orthologs in other *Pseudomonas* spp. is limited or null, we observed that they are frequently associated with genes encoding efflux pumps in the genome (some of them from the resistance nodulation division) suggesting a role in drug resistance. In addition to *nfxB* conservation, all residues affected by missense mutations were well conserved among *nfxB* orthologs (Figure S2). Thus, the *nfxB*/Cip^r^ system in combination with the luminescent reporter may be a valuable tool for studying mutational processes in *Pseudomonas* spp.

## Materials and Methods

### Bacterial strains and culture media

*P. aeruginosa* PAO1 wild-type (WT), and the isogenic *mutS, mutT, mutY* and *mutM* mutator strains were obtained from the University of Washington Genome Center [53]. Transposon insertion within the corresponding gene was confirmed by PCR analysis following the manufacturer’s instructions. Luria-Bertani (LB) or M9 minimal media were used [54]. To prepare inocula, bacteria were cultured on agar plates from frozen stocks and sub-cultured in liquid LB medium overnight with shaking at 240 rpm at 37°C.

### Estimation of spontaneous and mutagen-induced mutation rates

Mutation rates were determined by the modified Luria–Delbruck fluctuation test [55]. Independent cultures (10–30) were obtained as follows: *P. aeruginosa* WT and mutator cells from overnight cultures were inoculated in LB medium (∼300 cells/ml) and grown to exponential phase (∼1×10^9^ cells/ml). For 2-aminopurine (2AP) mutagenesis, the WT strain was cultured in LB medium as described above in the presence of 500 µg/ml 2AP. For cisplatin (CPT) and hydrogen peroxide (HP) mutagenesis, exponentially growing cultures of the WT strain in LB medium were diluted to 1×10^8^ cells/ml into M9 medium and incubated with 150 µg/mL CPT or 1.5 mM HP for 1 h at 37°C. Cells were then diluted 1000-fold into fresh LB medium and grown to exponential phase. After cultures reached late-exponential phase, aliquots from successive dilutions were plated onto LB agar to determine the number of viable cells and onto LB containing 0.5 or 1.0 µg/ml ciprofloxacin (Cip) to select resistant cells. Colonies were scored after 24–48 h. The Ma-Sandri-Sankar (MSS) maximum-likelihood method was applied to estimate the number of mutants (m) using the MSS algorithm [56]. Then, the mutation rate (µ) was calculated with the Luria–Delbruck equationµ = m/Nt, where Nt corresponds to the final number of cells in cultures [55]. Salvador program [57] was used to calculateµ and 95% confidence limits.

### Identification of *nfxB* mutants using a luminescence-based reporter

A luminescent reporter for detection of the transcriptional de-repression of the *mexCD-oprJ* operon was used to identify *nfxB* mutant cells [27]. Briefly, a DNA fragment (421-bp) containing the promoter region of *mexC* (160-bp) from *P. aeruginosa* PAO1 was fused to the *luxCDABE* operon, and this fusion was inserted into the attTn7 site of the *P. aeruginosa* chromosome using the pUC18-mini-Tn7T-Gm-*lux* vector [28]. Chromosomal insertions were confirmed by PCR as recommended in the published protocol. Luminescence was measured directly from Cip agar plates in a NightOWL LB 983 luminometer (Berhold Technologies). Colonies with enhanced luminescence exhibited a stable phenotype when picked up onto LB agar plates.

This reporter was employed to detect *nfxB* mutants in the *mutS* and *mutT* strains since a proportion of ciprofloxacin resistant (Cip^r^) colonies derived from these mutator strains was mutated in *nfxB* [27]. This proportion depended on the Cip concentration used for selection. When ∼1000 Cip^r^ colonies were analyzed, 30% and 10% of colonies with increased luminescence were observed at 0.5 and 1.0 µg/ml Cip for the *mutS* strain, respectively. At these Cip concentrations, the percentage of colonies with increased luminescence was 43% and 70% for the *mutT* strain. It was not necessary to use this reporter to identify *nfxB* mutants in the WT, *mutM* and *mutY* strains. For these strains, *nfxB* was mutated in all Cip^r^ colonies analyzed, which was consistent with the observation that 100% of ∼500 Cip^r^ colonies showed an increased luminescence phenotype (WT at 0.5 and 1.0 µg/ml Cip in the absence of mutagens, and at 0.5 µg/ml Cip in the presence of mutagens; *mutM* and *mutY* at 1.0 µg/ml Cip). Additionally, this reporter was used to sense the emergence of *nfxB* mutants in cells of the *mutT* strain exposed to sub-inhibitory Cip levels. Approximately 300 cells from 20 independent overnight cultures were applied onto LB agar plates containing 0.4 µg/ml Cip (6000 total cells). Luminescence of colonies was measured after 24 and 56 h of incubation.

### Analysis of the *nfxB* sequence in ciprofloxacin resistant mutants

*nfxB* (Genbank accession number: NC_002516.2; *Pseudomonas* Genome Database: PA4600, www.pseudomonas.com) was sequenced from colonies selected mainly at 0.5 µg/ml Cip for the WT (77%) and *mutS* (84%) strain. Additional sequences were obtained from colonies selected at 0.06, 0.6 and 1.0 µg/ml Cip for the WT strain and at 0.4 and 1.0 µg/ml Cip for the *mutS* strain. Cip^r^ colonies selected at 0.4 (31%), 0.5 (19%) and 1.0 (50%) µg/ml Cip were used for the *mutT* strain. 100% of colonies were selected at 1.0 µg/ml Cip for the *mutM* and *mutY* strains. In assays with mutagens, the WT strain was selected at 0.5 µg/ml Cip. For the *mutS* and *mutT* strains, *nfxB* mutants were identified among Cip^r^ populations using the chromosomal luminescent reporter described above. *nfxB* was sequenced in Cip^r^ colonies derived from independent cultures that were isolated in Cip agar plates to confirm its decreased Cip susceptibility.

For the WT strain, 0.5 µg/ml Cip was mainly used since the number of Cip^r^ colonies decreased ∼1000-fold at higher concentrations [27]. All analyzed Cip^r^ clones, selected at different Cip concentrations (0.06 to 1.0 µg/ml), were mutated in *nfxB* indicating that this gene may be the main mutation target independently of the Cip concentration used for selection. This was confirmed by inserting the luminescent reporter into the WT chromosome (see above). For the *mutS* and *mutT* strains, 0.5 and 1.0 µg/ml Cip were mainly employed, respectively, since the number of *nfxB* mutants was higher at these concentrations [27] (see above). It is important to note that we did not observe changes in the *nfxB* mutation spectra at the different Cip concentrations used for selection of the WT, *mutS* and *mutT* strains. Thus, we compiled the sequencing data obtained at different Cip concentrations for spectra analysis. *nfxB* mutation analysis for the *mutM* and *mutY* strains was only carried out at 1.0 µg/ml Cip. At this Cip concentration, all analyzed clones were mutated in *nfxB* and the Cip^r^ colonies showed an increased luminescence phenotype when the luminescent reporter was introduced into the chromosome of both strains (see above).

The entire open reading frame (564-bp) and the promoter region (160-bp) of the *nfxB* gene was amplified by colony PCR with primers NfxB1 (5'-AGCCATGACACACCCGACCG-3', complementary to the nucleotide sequence at positions 146-166 upstream of the ATG initiator codon) and NfxB2 (5'-TGCCATGCGGCGACGAGAGG-3', complementary to the nucleotide sequence at positions 42-62 downstream of the TGA stop codon). Amplified products were purified and sequenced with the NfxB1 primer by the DNA Sequencing Service of the University of Chicago. All mutations identified are shown in the Table S1.

The base substitution spectra obtained from the WT, *mutS* and *mutT* strains were statistically analyzed with iMARS [25]. iMARS was used to examine individual spectra for significant hotspots to distinguish true mutation hotspots from potential randomly mutable sites. The approach used by iMARS for identification of hotspots within individual spectra is based on that described by Tarone [25]. To identify underlying patterns of target-sequence context, the relative dinucleotide mutability (RDM) scores for the 12 possible dinucleotides within each spectrum were calculated by the nearest neighbor analysis used by iMARS (see Table S3). In addition, the mutation sequence context was examined by analyzing the five nucleotides upstream and downstream of the mutational site (see Table S1).

To determine the spontaneous *rpoB* mutation spectrum from the WT strain, cells were cultured as described above and plated onto LB agar containing 100 µg/ml rifampicin. The region corresponding to cluster I and II of *rpoB* was PCR amplified and sequenced in 44 independent rifampicin resistant clones using the primers RpoB1 (5'- AATGGCCGAGAACCAGTTCCG-3') and RpoB2 (5'- AAGCCTGGGCGATGACGTGG-3').

### NfxB tertiary structure homology modeling

A tridimensional homology model of NfxB was made using MODELLER 9v8 [20]. Since BLAST and PSI-BLAST retrieved homologous sequences with known three-dimensional structure but a low identity to NfxB, the fold recognition software pGenTHREADER [58] (http://bioinf.cs.ucl.ac.uk/psipred/) was used to identify a suitable template structure and to obtain a target-template sequence alignment. The top hit was the TetR-like transcriptional repressor LfrR from *Mycobacterium Smegmatis* in complex with proflavine (PDB code: 2V57). This repressor, which is also involved in Cip resistance [21], was used as the model template. The predicted NfxB amino acid sequence has an identity of 22% with LfrR.

Initial models were built with automodel class. The model with the lowest value of the MODELLER objective function was picked. Evaluation of the model using QMEAN [59] revealed a loop of poor quality (residues 130 to 140) that corresponded to a gap in the alignment. This region was further refined using the loop routine of MODELER [20]. Final refinement was performed generating 50 additional models, followed by selection of the best model based on the global QMEAN score [59]. The resulting model had a good quality and a strong reliability as indicated by a QMEAN score of 0.7 (range 0–1; where 0 is worst and 1 best). Secondary assessment of NfxB model was determined using the knowledge-based method STRIDE [60] and DSSP [61], [62] that uses hydrogen bonded patterns. No significant differences arose from these two methods. Electrostatic Surface potential was calculated with APBS [23]. The final model was represented with VMD (Figure 2A) [63].

Secondary structure of NfxB was predicted using JPred [22] (Figure S1). Comparison of the NfxB secondary structure predicted by JPred with that experimentally determined for LfrR revealed a strong correlation in the alignment supporting the selection of LfrR as a template for NfxB structure modeling. In addition, the localization of a high proportion of missense mutations in the putative DNA-binding and dimerization regions and the predicted effects of these changes on NfxB function also supported the obtained model.

## Supporting Information

Figure S1 **Secondary structure analysis of NfxB.** The secondary NfxB structure was predicted from amino acid sequence using JPred. H: Helix; S: Bend; T: Turn.
(TIF)

Figure S2 ***nfxB*** **sequence conservation in** ***Pseudomonas*** **spp.** Sequence alignment of NfxB from *P. aeruginosa* PAO1 (PA4600) to some of its putative orthologs from other strains and species. *P. aeruginosa* M18 (PAM18); *P. aeruginosa* UCBPP-PA14 (PA14); *P. aeruginosa* PA7 (PSPA7); *P. fluorescens* Pf0-1 (Pfl01); *P. entomophila* L48 (PSEEN); *P. putida* GB-1 (PputGB1); *P. putida* KT2440 (PP); *P. stutzeri* DSM 10701 (PSJM300); *P. syringae* pv. phaseolicola 1448A (PSPPH); *P. syringae* pv. syringae B728a 89 (Psyr); *P. syringae* pv. tomato DC3000 (PSPTO) and *P. fulva* 12-X (Psefu). Sequences were aligned using ClustalW (www.ebi.ac.uk). All amino acids found to undergo missense mutations among the Cip^r^ PAO1 clones analyzed in this work are coloured in red. Amino acids found to undergo missense mutations among quinolone resistant or *mexCD-oprJ* overexpressing *P. aeruginosa* clones in previous studies are coloured in cyan. Residues detected both in this work and previous studies are indicated in orange.
(TIF)

Table S1 **Mutations in** ***nfxB.***
(DOC)

Table S2 **Percentage of each** ***nfxB*** **mutation^a^.**
(DOC)

Table S3 **RDM scores^a^.**
(DOC)

Table S4 **Mutations in** ***rpoB^a^*** **.**
(DOC)
