# Supplementary figures and images for: nfxB as a Novel Target for Analysis of Mutation Spectra in Pseudomonas aeruginosa
Source: PLoS One. 2013 Jun 7;8(6):e66236. doi: 10.1371/journal.pone.0066236 (PMC3676378; doi:10.1371/journal.pone.0066236)

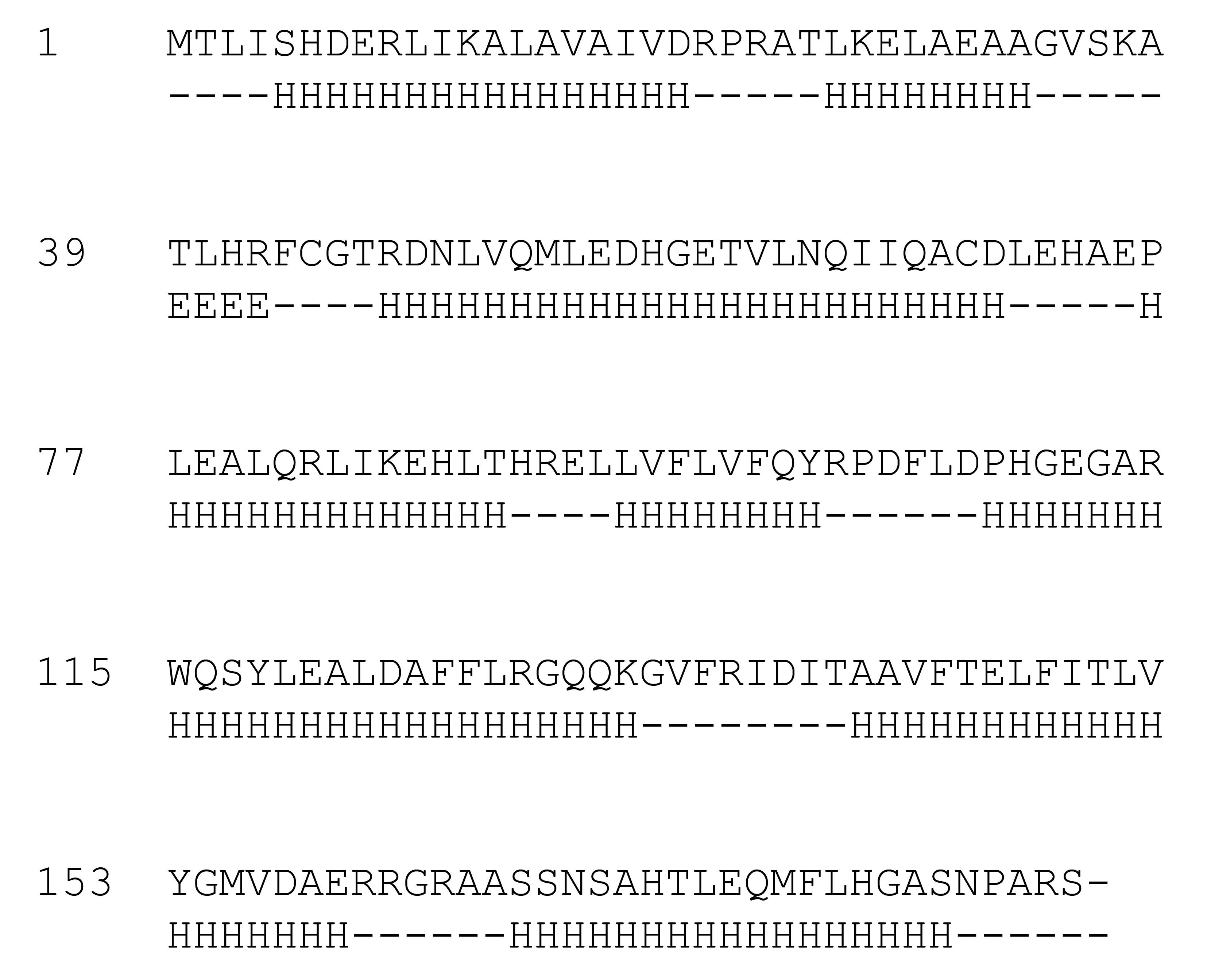

Supplement: Figure S1 — Secondary structure analysis of NfxB. The secondary NfxB structure was predicted from amino acid sequence using JPred. H: Helix; S: Bend; T: Turn. (TIF) [file pone.0066236.s001.tif]

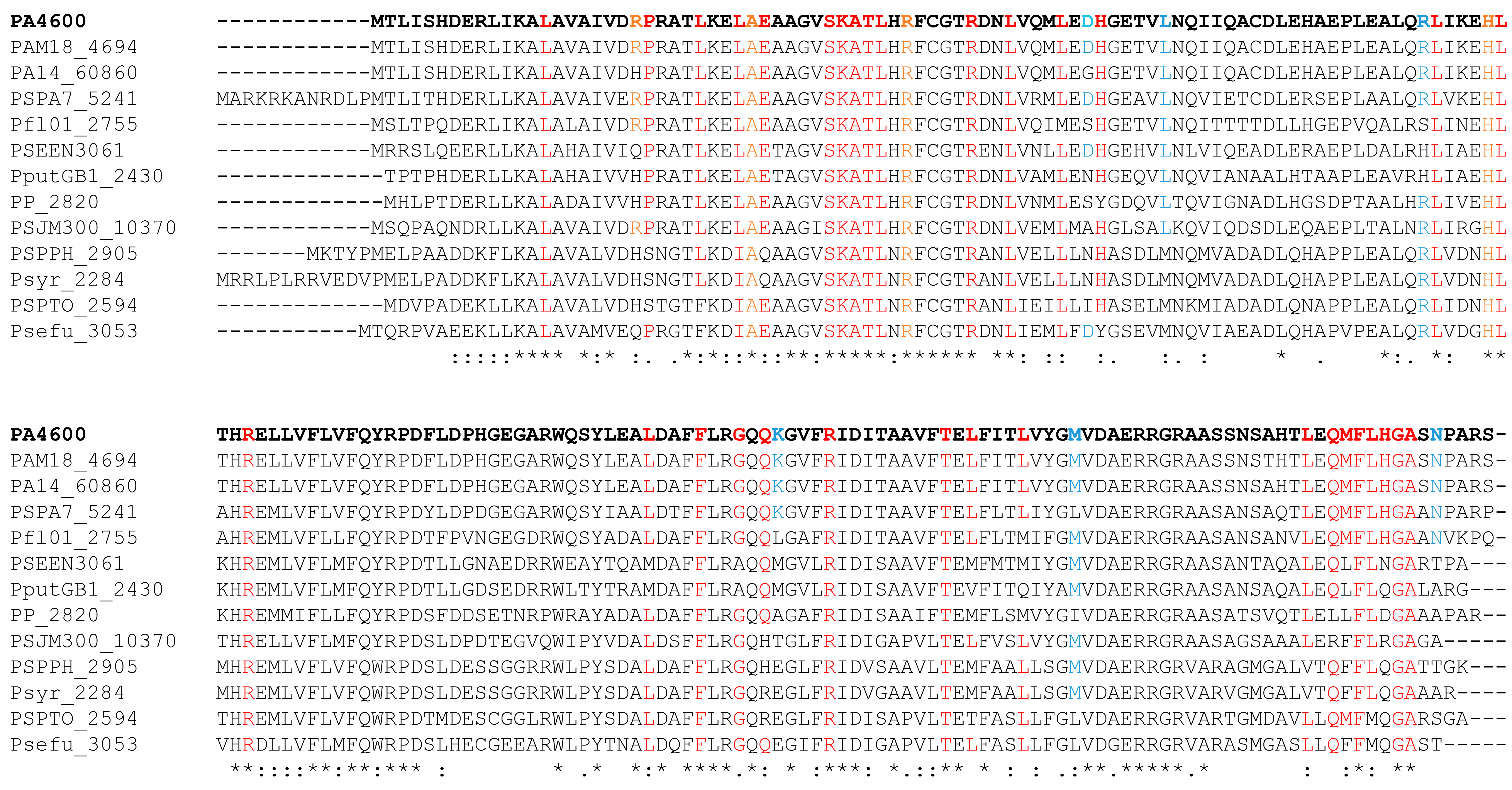

Supplement: Figure S2 — nfxB sequence conservation in Pseudomonas spp. Sequence alignment of NfxB from P. aeruginosa PAO1 (PA4600) to some of its putative orthologs from other strains and species. P. aeruginosa M18 (PAM18); P. aeruginosa UCBPP-PA14 (PA14); P. aeruginosa PA7 (PSPA7); P. fluorescens Pf0-1 (Pfl01); P. entomophila L48 (PSEEN); P. putida GB-1 (PputGB1); P. putida KT2440 (PP); P. stutzeri DSM 10701 (PSJM300); P. syringae pv. phaseolicola 1448A (PSPPH); P. syringae pv. syringae B728a 89 (Psyr); P. syringae pv. tomato DC3000 (PSPTO) and P. fulva 12-X (Psefu). Sequences were aligned using ClustalW (www.ebi.ac.uk). All amino acids found to undergo missense mutations among the Cipr PAO1 clones analyzed in this work are coloured in red. Amino acids found to undergo missense mutations among quinolone resistant or mexCD-oprJ overexpressing P. aeruginosa clones in previous studies are coloured in cyan. Residues detected both in this work and previous studies are indicated in orange. (TIF) [file pone.0066236.s002.tif]
