# Supplementary material for: nfxB as a Novel Target for Analysis of Mutation Spectra in Pseudomonas aeruginosa
Source: PLoS One. 2013 Jun 7;8(6):e66236. doi: 10.1371/journal.pone.0066236 (PMC3676378; doi:10.1371/journal.pone.0066236)
Supplement: Table S1 — Mutations in nfxB. (DOC) [file pone.0066236.s003.doc]

Table S1. Mutations in *nfxB*

| bp change^a^ | site (bp) | Sequence context^b^ | protein change^c^ | Number of mutation occurrence for: | | | | | | | |
| --- | --- | --- | --- | --- | --- | --- | --- | --- | --- | --- | --- |
|  |  |  |  | WT | *mutS* | *mutT* | *mutY* | *mutM* | 2AP | CPT | HP |
| AT>GC | 41  77  86  119  149  161  248  260  263  365  377  440  452  518  533  562 | GGCGC**T**GGCAG  GACGC**T**GAAGG  GGAAC**T**GGCCG  CACCC**T**GCACC  CAACC**T**GGTGC  GATGC**T**CGAGG  GCGCC**T**GATCA  GGAAC**A**CCTCA  ACACC**T**CACCC  AGCGC**T**GGACG  CTTCT**T**CCTGC  CACCG**A**ACTGT  CACCC**T**GGTCT  TACCC**T**GGAGC  GTTCC**T**CCATG  GCTCC**T**GACCC | L14P  L26P  L29P  L40P  L50P  L54P  L83P  H87R  L88 P  L122P  F126S  L147P  L151P  L173P  L178P  Stop188R | 0  0  0  0  0  1  0  2  0  0  0  1  0  0  0  0 | 2  3  6  4  2  0  3  6  1  0  1  3  5  1  1  2 | 0  0  0  0  0  0  0  1  0  0  0  0  0  0  0  0 | 0  0  0  0  0  0  0  0  0  0  0  0  0  0  0  0 | 0  0  0  0  0  0  0  0  0  0  0  0  0  0  0  0 | 0  0  0  0  1  0  0  0  0  1  0  0  0  0  0  0 | 0  0  0  0  0  0  0  0  0  0  0  0  0  0  0  0 | 0  0  0  0  0  0  0  0  0  0  0  0  1  0  0  0 |
| GC>AT | 65  88  91  107  113  116  124  125  140  154  190  199  271  298  346  385  388  406  523  538  539 | CCGCC**C**GCGAG  AACTG**G**CCGAG  TGGCC**G**AGGCG  CGTAA**G**CAAGG  CAAGG**C**CACCC  GGCCA**C**CCTGC  TGCAC**C**GCTTC  GCACC**G**CTTCT  CACGC**G**GGACA  TGGTG**C**AGATG  TGAAC**C**AGATC  TCATC**C**AGGCC  CCCAC**C**GCGAG  TATTC**C**AGTAC  GCTGG**C**AGTCC  TGCGC**G**GACAG  GCGGA**C**AGCAG  TGTTT**C**GCATC  TGGAG**C**AGATG  TCCAT**G**GCGCC  CCATG**G**CGCCT | P22L  A30T  E31K  S36N  A38V  T39I  R42C  R42H  R47Q  Q52Stop  Q64Stop  Q67Stop  R91C  Q100Stop  Q116Stop  G129R  Q130Stop  R136C  Q175Stop  G180S  G180D | 4  0  1  2  0  0  1  4  0  0  1  0  0  0  1  0  0  0  2  0  1 | 2  1  0  0  1  1  0  0  1  1  0  0  1  0  0  2  0  2  0  1  3 | 0  0  0  0  0  0  0  0  0  0  0  0  0  0  0  0  0  0  0  0  0 | 0  0  0  0  0  0  0  0  0  0  0  0  0  0  0  0  0  0  0  0  0 | 0  0  0  0  0  0  0  0  0  0  0  0  0  1  0  0  0  0  0  0  0 | 3  0  0  0  0  0  2  0  0  0  0  1  0  0  1  0  0  0  0  0  0 | 0  0  0  0  0  0  0  0  0  0  0  0  0  0  0  0  0  0  0  0  0 | 1  0  0  1  0  0  0  0  0  0  0  0  0  0  0  0  1  0  0  0  0 |
| AT>TA | 41  119  149 | GGCGC**T**GGCAG  CACCC**T**GCACC  CAACC**T**GGTGC | L14Q  L40Q  L50Q | 3  1  1 | 0  0  0 | 0  0  0 | 0  0  0 | 0  0  0 | 0  0  0 | 2  0  0 | 0  0  0 |
| AT>CG | 41  106  109  110  115  119  170  365  392  433  524  527  530  536  564 | GGCGC**T**GGCAG  GCGTA**A**GCAAG  TAAGC**A**AGGCC  AAGCA**A**GGCCA  AGGCC**A**CCCTG  CACCC**T**GCACC  GGACC**A**CGGAG  AGCGC**T**GGACG  GCAGA**A**AGGCG  TGTTC**A**CCGAA  GGAGC**A**GATGT  GCAGA**T**GTTCC  GATGT**T**CCTCC  CCTCC**A**TGGCG  TCCTG**A**CCCTC | L14R  S36R  K37Q  K37T  T39P  L40R  H57P  L122R  Q131P  T145P  Q175P  M176R  F177C  H179P  Stop188C | 0  0  0  0  18  1  0  0  0  0  0  0  0  0  0 | 0  0  0  0  0  0  0  0  0  0  0  1  0  0  0 | 0  6  3  1  0  0  3  2  2  3  3  0  1  4  0 | 0  0  0  0  0  0  0  0  0  0  0  0  0  0  0 | 0  0  0  0  2  0  0  0  0  0  0  0  0  0  0 | 0  0  0  0  0  0  0  0  0  0  0  0  0  0  0 | 1  0  0  0  1  0  0  0  0  0  0  0  0  0  1 | 0  0  0  0  3  0  0  0  0  0  0  0  0  0  0 |
| GC>TA | 82  91  175  214  223  232  256  274  358  475  484 | TGAAG**G**AACTG  TGGCC**G**AGGCG  ACGGA**G**AGACC  GACCT**G**GAGCA  ATGCC**G**AGCCT  CTCTG**G**AGGCG  ATCAA**G**GAACA  ACCGC**G**AGCTG  ACCTG**G**AAGCG  ATGCG**G**AACGT  GTCGC**G**GACGG | E28Stop  E31Stop  E59Stop  E72Stop  E75Stop  E78Stop  E86Stop  E92Stop  E120Stop  E159Stop  G162Stop | 1  0  0  0  0  2  0  0  0  0  0 | 0  0  0  0  0  0  0  0  0  0  0 | 0  0  0  0  0  0  0  0  0  0  0 | 0  1  1  0  0  0  1  4  1  1  1 | 0  0  0  1  0  0  0  1  0  0  0 | 0  0  0  0  0  0  0  0  0  0  0 | 0  0  0  0  1  0  0  1  0  0  0 | 0  0  0  0  0  0  0  0  0  0  0 |
| GC>CG | 62  88  125  459  541 | CGACC**G**CCCGC  AACTG**G**CCGAG  GCACC**G**CTTCT  GTCTA**C**GGCAT  GAACT**G**TTCAT | R21P  A30P  R42P  Y153Stop  A181P | 0  0  1  0  2 | 0  1  0  0  0 | 0  0  0  0  0 | 0  0  0  0  0 | 2  0  0  1  0 | 0  0  0  0  0 | 0  0  0  0  0 | 0  0  0  0  0 |
| 1-bp insertion | 5*insC*  40*insC*  102*insC*  163*insG*  182*insT*  443*insG*  496*insA*  519*insG* | GATGA**C**CCTGA  AGGCG**C**TGGCA  GCCGG**C**GTAAG  TGCTC**G**AGGAC  GACCG**T**ACTGA  ACTGT**T**CATCA  CGGCC**A**GCTCC  ACCCT**G**GAGCA | frameshift*  frameshift  frameshift  frameshift  frameshift  frameshift  frameshift  frameshift | 0  1  1  1  1  1  0  1 | 1  0  0  0  0  0  0  0 | 0  0  0  0  0  0  0  0 | 0  0  0  0  0  0  0  0 | 0  0  0  0  0  0  0  0 | 0  0  0  0  0  0  0  0 | 0  0  0  0  0  0  1  0 | 0  0  0  0  0  0  0  0 |
| 1-bp deletion | 89*delC*  98*delC*  115*delA*  120*delG*  136*delA*  144*delC*  172*delG*  184*delC*  308*delC*  317*delT*  373*delT*  464*delT*  475*delG*  515*delC*  539*delG* | AACTG**G**CCGAG  GGCGG**C**CGGCG  AGGCC**A**CCCTG  ACCCT**G**CACCG  GCGGC**A**CGCGG  CGGGA**C**AACCT  CCACG**G**AGAGA  CCGTA**C**TGAAC  CCGCC**C**GGACT  CTTCC**T**CGACC  ACGCC**T**TCTTC  CGGCA**T**GGTCG  ATGCG**G**AACGT  GCATA**C**CCTGG  CCATG**G**CGCCT | frameshift*  frameshift*  frameshift*  frameshift*  frameshift*  frameshift*  frameshift*  frameshift*  frameshift  frameshift  frameshift  frameshift  frameshift  frameshift  frameshift | 1  1  1  0  1  0  0  1  1  1  0  1  0  1  3 | 0  0  0  0  0  0  1  0  0  0  1  0  0  0  0 | 0  0  0  0  0  0  0  0  0  0  0  0  0  0  0 | 0  0  0  0  0  0  0  0  0  0  0  0  0  0  0 | 0  0  0  0  0  1  0  0  0  0  0  0  0  0  0 | 0  0  0  0  0  0  0  0  0  0  0  0  0  0  0 | 0  0  0  1  0  0  0  0  0  0  0  0  1  0  0 | 0  0  0  0  0  0  0  0  0  0  0  0  0  0  0 |
| 1bp<del <15bp | 44-45  105-107  136-147  161-172  184-195  194-205  195-205  215-227  232-241  323-335  341-351  445-456  463-464  520-530  537-538  537-547  538-549 |  | frameshift  36*del*  46-49*del*  54-58*del*  62-66*del*  65-68*del*, C69S  frameshift  frameshift*  frameshift  frameshift  frameshift  49-52*del*  frameshift  frameshift  frameshift  frameshift  frameshift | 1  1  1  0  0  1  1  1  0  1  0  1  0  1  1  0  1 | 0  0  0  0  0  0  0  0  0  0  0  0  0  0  0  0  0 | 0  0  0  0  0  0  0  0  0  0  3  0  0  0  0  0  0 | 0  0  0  0  0  0  0  0  0  0  0  0  0  0  0  0  0 | 0  0  0  0  0  0  0  0  0  0  0  0  0  0  0  0  0 | 0  0  0  0  0  0  0  0  0  0  0  0  0  0  0  0  0 | 0  0  0  0  1  0  0  0  0  0  0  0  0  0  0  0  0 | 0  0  0  1  0  0  0  0  1  0  0  0  1  0  0  1  0 |
| 15bp<del <150bp | 39upstream-3  45-89  297-404  364-471  367-481  439-465 |  | 1*del*  16-30*del*  frameshift  122-157*del*  frameshift  147-151*del* | 0  1  1  1  1  0 | 0  0  0  0  0  0 | 0  0  0  0  0  0 | 0  0  0  0  0  0 | 0  0  0  0  0  0 | 1  0  0  0  0  0 | 0  0  0  0  0  0 | 0  0  0  0  0  1 |
| del>150 bp | 30upstream-182  38-339  127-402  255-484 |  | frameshift  frameshift  44-134*del*  frameshift | 0  1  1  0 | 0  0  0  0 | 0  0  0  0 | 0  0  0  0 | 0  0  0  1 | 0  0  0  0 | 1  0  0  0 | 0  0  0  0 |
| Duplication | 4-5  79-90  152-295  376-381  417-438  503-504  526-529 |  | frameshift*  27-30*dup*  52-98*dup*  26-27*dup*  frameshift  frameshift  frameshift | 1  1  1  1  1  1  1 | 0  0  0  0  0  0  0 | 0  0  0  0  0  0  0 | 0  0  0  0  0  0  0 | 0  0  0  0  0  0  0 | 0  0  0  0  0  0  0 | 0  0  0  0  0  0  0 | 0  0  0  0  0  0  0 |
| Total |  |  |  | 93 | 61 | 32 | 10 | 10 | 10 | 12 | 12 |

^a^*nfxB* was sequenced from independent colonies derived from the WT, *mutS*, *mutT*, *mutM* and *mutY* strains and the WT strain treated with mutagens 2-aminopurine (2AP), cisplatin (CPT) and hydrogen peroxide (HP). ^b^Sequences surrounding the mutational site are showed for all base substitutions and deletions and insertions of 1-bp. The nucleotide undergoing mutation is indicated in bold. The A, T, C and D nucleotides are colored in purple, green, cian and yellow, respectively. ^c^Frameshifts that cause premature stop codons are indicated by an asterisk (*). The main protein changes are indicated for >1-bp deletions and duplications.
