# Supplementary material for: nfxB as a Novel Target for Analysis of Mutation Spectra in Pseudomonas aeruginosa
Source: PLoS One. 2013 Jun 7;8(6):e66236. doi: 10.1371/journal.pone.0066236 (PMC3676378; doi:10.1371/journal.pone.0066236)
Supplement: Table S2 — Percentage of each nfxB mutationa. (DOC) [file pone.0066236.s004.doc]

Table S2. Percentage of each *nfxB* mutation^a^

| Strain/  mutagen | AT>GC | GC>AT | AT>TA | AT>CG | GC>TA | GC>CG | 1-bp  insertion | 1-bp  deletion | >1-bp  deletion | duplication |
| --- | --- | --- | --- | --- | --- | --- | --- | --- | --- | --- |
| WT | 4 | 18 | 5 | 20 | 3 | 3 | 6 | 13 | 18 | 8 |
| *mutS* | 66 | 26 | 0 | 2 | 0 | 2 | 2 | 3 | 0 | 0 |
| *mutT* | 3 | 0 | 0 | 88 | 0 | 0 | 0 | 0 | 9 | 0 |
| *mutY* | 0 | 0 | 0 | 0 | 100 | 0 | 0 | 0 | 0 | 0 |
| *mutM* | 0 | 10 | 0 | 20 | 20 | 30 | 0 | 10 | 10 | 0 |
| 2AP | 20 | 70 | 0 | 0 | 0 | 0 | 0 | 0 | 10 | 0 |
| CPT | 0 | 0 | 17 | 25 | 17 | 0 | 8 | 17 | 17 | 0 |
| HP | 8 | 25 | 0 | 25 | 0 | 0 | 0 | 0 | 42 | 0 |

^a^The percentage of each mutation type detected in *nfxB* from the WT and mutator strains (*mutS*, *mutT*, *mutY* and *mutM*), and the WT strain after exposure to 2-aminopurine (2AP), cisplatin (CPT) and hydrogen peroxide (HP) is shown. Percentage refers to the occurrence of each observed mutation as a function of the total number of mutations detected for each strain or mutagen treatment.
