# Supplementary material for: nfxB as a Novel Target for Analysis of Mutation Spectra in Pseudomonas aeruginosa
Source: PLoS One. 2013 Jun 7;8(6):e66236. doi: 10.1371/journal.pone.0066236 (PMC3676378; doi:10.1371/journal.pone.0066236)
Supplement: Table S3 — RDM scoresa. (DOC) [file pone.0066236.s005.doc]

Table S3. RDM scores^a^

| Dinucleotide | Strain | | |
| --- | --- | --- | --- |
|  | WT | *mutS* | *mutT* |
| AA | 0.0 | 0.0 | 5.3 |
| AG | 0.6 | 0.0 | 3.6 |
| AC | 4.7 | 1.7 | 1.7 |
| AT | 0.0 | 0.4 | 1.6 |
| GA | 0.9 | 0.0 | 0.0 |
| GG | 0.9 | 1.9 | 0.0 |
| GC | 1.8 | 1.0 | 0.0 |
| GT | 0.0 | 0.0 | 0.0 |
| CA | 5.5 | 1.6 | 4.4 |
| CG | 2.0 | 1.2 | 0.0 |
| CC | 1.0 | 0.8 | 0.0 |
| CT | 1.8 | 7.4 | 0.5 |
| TA | 0.0 | 0.0 | 0.0 |
| TG | 1.8 | 8.5 | 0.5 |
| TC | 0.2 | 1.2 | 0.2 |
| TT | 0.0 | 0.6 | 0.6 |

^a^The relative dinucleotide mutability (RDM) scores were calculated using iMARS as depicted in Material and Methods
