# Supplementary material for: nfxB as a Novel Target for Analysis of Mutation Spectra in Pseudomonas aeruginosa
Source: PLoS One. 2013 Jun 7;8(6):e66236. doi: 10.1371/journal.pone.0066236 (PMC3676378; doi:10.1371/journal.pone.0066236)
Supplement: Table S4 — Mutations in rpoBa . (DOC) [file pone.0066236.s006.doc]

Table S4. Mutations in *rpoB^a^*

| bp change | Site (bp) | Number of mutants | Percentage |
| --- | --- | --- | --- |
| AT>GC | 1549  1553  1562  1592 | 1  1  14  2 | 41 |
| GC>AT | 1580  1591  1607  1736 | 1  5  6  1 | 30 |
| AT>TA | 1553  1562  1592 | 1  2  8 | 25 |
| GC>TA | 1591 | 1 | 2 |
| 1bp<del<15bp | 1550-1561 | 1 | 2 |
| Total |  | 44 | 100 |

^a^*rpoB* was sequenced from rifampicin resistant clones derived from the WT strain (see Materials and Methods).
